# A novel nomogram for predicting early postoperative cerebral desaturation events after congenital heart surgery

**DOI:** 10.3389/fsurg.2026.1808700

**Published:** 2026-04-23

**Authors:** Siyuan Wang, Weihong Xu, Bin Ji, Menglian Sun, Jianhu Huang, Jiwen Sun, Nanping Shen

**Affiliations:** 1 Department of Anesthesiology, Shanghai Children’s Medical Center, Shanghai Jiao Tong University School of Medicine, Shanghai, China; 2 Department of Thoracic and Cardiovascular Surgery, Shanghai Children’s Medical Center, Shanghai Jiao Tong University School of Medicine, Shanghai, China; 3 Department of Nursing, Shanghai Children’s Medical Center, Shanghai Jiao Tong University School of Medicine, Shanghai, China

**Keywords:** cardiac surgery, cardiopulmonary bypass, cerebral desaturation, near-infrared spectroscopy, perioperative neuroprotection, risk prediction nomogram

## Abstract

**Background:**

Infants undergoing congenital heart surgery with cardiopulmonary bypass (CPB) are at high risk for perioperative neurological injury, and early postoperative cerebral desaturation events (CDEs) remain common. Reliable tools for individualized risk stratification are lacking. This study aims to develop and internally validate a nomogram for predicting early postoperative CDEs in infants undergoing congenital heart surgery with CPB.

**Methods:**

This retrospective cohort study included 397 infants aged >1 month and ≤1 year who underwent elective congenital heart surgery with CPB. Patients were randomly divided into a development cohort (*n* = 277) and a validation cohort (*n* = 120). Early postoperative CDEs were assessed using near-infrared spectroscopy monitoring at cardiac intensive care unit admission. Prespecified perioperative variables measured before or during CPB were entered into a multivariable logistic regression model to construct a nomogram. Model performance was evaluated using discrimination, calibration, and decision curve analysis, with internal validation.

**Results:**

Early postoperative CDEs occurred in 16.88% of the overall cohort, with comparable incidences in the development (18.1%) and validation (14.2%) cohorts. The final model included body surface area, RACHS-1 category, CPB duration, hematocrit during CPB, and CPB temperature strategy. The nomogram demonstrated good discrimination in the development cohort [C-statistic 0.798, 95% confidence interval (CI) 0.729–0.857] and the validation cohort (0.767, 95% CI 0.629–0.886), with satisfactory calibration. The decision curve analysis suggested potential clinical usefulness across clinically relevant threshold probabilities.

**Conclusions:**

An internally validated, infant-specific nomogram based on routinely available perioperative variables was developed to predict early postoperative cerebral desaturation following congenital heart surgery with CPB. External validation is required before clinical implementation.

## 1 Introduction

Congenital heart disease (CHD) is among the most common birth defects, affecting more than 2 million children in China (1). Although advances in surgical techniques and perioperative management have markedly improved survival, long-term morbidity—particularly adverse neurodevelopmental outcomes—remains a major concern (2). Previous studies indicate that approximately 40%–50% of children who survive CHD surgery experience some degree of neurodevelopmental impairment (3, 4). Infants undergoing cardiac surgery with cardiopulmonary bypass (CPB) are especially vulnerable to perioperative neurological injury, a risk closely linked to disturbances in cerebral oxygen balance (5, 6).

Several modalities are available for assessing perioperative brain injury or subsequent neurodevelopmental sequelae, including magnetic resonance imaging, circulating biomarkers, and standardized neurodevelopmental assessments (7, 8). However, these approaches primarily detect already-established structural injury or delayed functional deficits. In contrast, near-infrared spectroscopy (NIRS) enables non-invasive, continuous monitoring of regional cerebral oxygen saturation (rScO₂), facilitating early identification of cerebral desaturation events (CDEs) during and immediately after cardiac surgery (9, 10).

In infants undergoing CPB-assisted cardiac surgery, CDEs are common and have been associated with adverse clinical outcomes such as prolonged hospitalization (11) and worse long-term neurodevelopment (12). Given these associations, timely perioperative identification and management of CDEs are clinically meaningful. However, widespread implementation of NIRS is limited by practical constraints such as challenges with sensor fixation, equipment cost, and potential variability in measurement performance related to skin pigmentation (3, 10). Beyond these technical hurdles, high equipment and sensor costs cause significant regional disparities in NIRS availability, particularly in resource-limited settings, as highlighted by a recent national survey in China (13). Furthermore, NIRS is inherently a reactive monitor, alerting clinicians only after a CDE has occurred. Therefore, a proactive and cost-effective prediction model using routine perioperative variables is urgently needed. Such an early warning framework can identify high-risk infants in advance, optimizing the allocation of expensive monitoring resources and facilitating preemptive neuroprotection.

Growing evidence suggests that several perioperative factors may influence cerebral oxygen balance by altering cerebral perfusion, oxygen delivery, and metabolic demand (14), including age, surgical duration, cerebral perfusion, hematocrit, and CPB temperature (15–18). However, most of these factors have been investigated either in heterogeneous pediatric populations or as isolated intraoperative associations and have not been integrated into models for individualized risk assessment. As a result, in routine clinical practice, accurate prediction of postoperative CDEs in infants undergoing CPB remains challenging.

To date, no well-validated model has been established to estimate the risk of postoperative CDEs in this specific population. To fill this research gap, the present study aims to identify perioperative predictors of postoperative CDEs in infants and to develop and internally validate a nomogram for individualized risk estimation.

## 2 Methods

### 2.1 Ethics

This single-center retrospective observational study was approved by the Institutional Review Board of Shanghai Children's Medical Center (Approval No. SCMCIRB-K2023029-1). The requirement for informed consent was waived because of the retrospective nature of the study. All procedures were conducted in accordance with the Declaration of Helsinki. The study was reported in compliance with the STROBE guidelines for observational studies.

### 2.2 Study population

**Figure 1:**
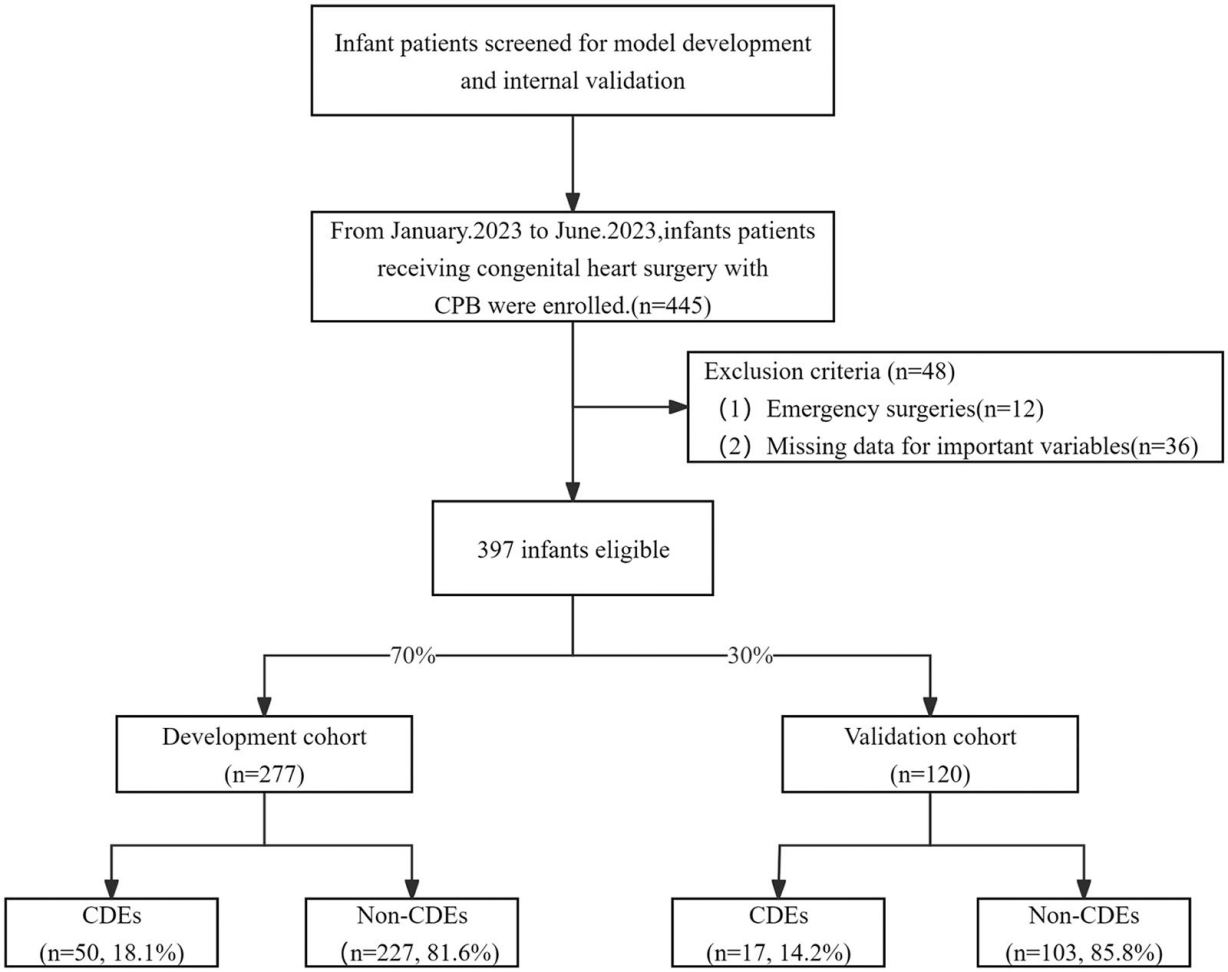
Flow chart of the patient selection process.

Between 1 January 2023 and 1 June 2023, we retrospectively reviewed consecutive infants at the Shanghai Children's Medical Center. The inclusion criteria were: (1) congenital heart disease treated with cardiac surgery requiring cardiopulmonary bypass (CPB); (2) age >1 month and ≤1 year. The exclusion criteria were: (1) emergency surgery or preoperative critical conditions; (2) severe preoperative neurological or other systemic diseases; (3) >20% missing clinical data; and (4) postoperative extracorporeal membrane oxygenation support. Following these criteria, the eligible patients were randomly assigned to a development cohort (*n* = 277) and a validation cohort (*n* = 120) at a ratio of 7:3. The detailed patient selection and allocation process is presented in Figure 1.

### 2.3 Data collection and candidate predictors

Demographic, preoperative, intraoperative, and early postoperative data were extracted from the institutional electronic medical record system. Based on clinical relevance and prior literature, we prespecified 19 perioperative variables measured before or during CPB as candidate predictors for model development. These included demographic characteristics (age, sex, body weight, and body surface area), preoperative physiological parameters (peripheral oxygen saturation, mean arterial pressure, and hematocrit), surgical characteristics [anesthesia time, surgical position, history of cardiac surgery, surgical complexity assessed by the Risk Adjustment for Congenital Heart Surgery-1 (RACHS-1) category (19) and ASA grade (20)], and CPB-related variables (lowest CPB temperature, CPB duration, and intraoperative hematocrit that measured, after a modified ultrafiltration, a protocolized and consistently recorded time point at our center.).

Only variables available before the prediction time point and recorded with sufficient completeness were considered candidate predictors for multivariable modeling. Some potentially relevant perioperative factors were not entered into model development because they were not consistently documented in this retrospective cohort [e.g., the lowest intraoperative hematocrit and hematocrit before cardiac intensive care unit (CICU) admission] or showed limited variability under standardized institutional management (e.g., intraoperative respiratory support and anesthetic regimens).

### 2.4 Outcomes and definitions

The primary outcome was an early postoperative cerebral desaturation event (CDE) at admission to the CICU. Upon CICU admission, rScO₂ values were continuously recorded, and the mean rScO₂ during the first 5 min after admission was calculated. Regional cerebral oxygen saturation (rScO₂) was continuously monitored using near-infrared spectroscopy (INVOS™ 5100C, Medtronic, USA). Sensors were placed bilaterally on the forehead according to the manufacturer's instructions. We adopted a prespecified operational definition based on established clinical intervention algorithms (20). Specifically, a CDE was defined as either an absolute mean rScO_2_ < 50% within the first 5 min of admission or a relative decrease of >20% from the preoperative baseline. These thresholds have been previously validated as significant predictors of major morbidity and adverse neurodevelopmental outcomes in children undergoing congenital heart surgery (21).

### 2.5 Statistical analysis

Continuous variables were assessed for normality using the Shapiro–Wilk test. Normally distributed data are presented as mean ± standard deviation and were compared using the independent-samples *t*-test, while non-normally distributed data are expressed as median (interquartile range) and were compared using the Mann–Whitney *U* test. Categorical variables are reported as counts (percentages) and were analyzed using the chi-square test or Fisher's exact test, as appropriate. Given that variables were extracted from the electronic medical record system, data completeness was generally high. Patients with >20% missing clinical or rScO₂ data were excluded, and the remaining sporadic missing values were handled using multiple imputation with chained equations.

As this was a retrospective cohort study, no formal *a priori* sample size calculation was performed; the sample size was determined by the total number of eligible infants (*N* = 397) available during the study period. Eligible patients were randomly divided into a development cohort (*n* = 277) and a validation cohort (*n* = 120) at a 7:3 ratio, utilizing a fixed random seed to ensure reproducibility. Given the overall event rate of approximately 16.9% (yielding roughly 47 events in the development cohort), model development was strictly guided by the events-per-variable (EPV) principle to minimize the risk of overfitting. To adhere to this principle and maintain an acceptable EPV ratio, candidate predictors were systematically filtered. Initially, potential variables were screened using univariable analyses, and those demonstrating statistical significance were further evaluated via univariable logistic regression. Subsequently, only the remaining significant predictors, which also possessed clinical relevance, were entered into the multivariable logistic regression model. Notably, to avoid sparse data bias caused by numerous specific surgical procedures, the RACHS-1 score was utilized as a standardized proxy for surgical complexity. Given the rarity of cases in the highest risk categories, this score was dichotomized into two groups (<3 and ≥3) to maintain statistical stability. A backward stepwise selection procedure was then applied to identify the final independent predictors for postoperative CDEs, yielding a parsimonious model that was visualized as a nomogram.

Model performance was rigorously evaluated in both cohorts. Discrimination was quantified using the area under the receiver operating characteristic curve (AUROC). Calibration was assessed using calibration plots, the Hosmer–Lemeshow goodness-of-fit test, the Brier score, calibration-in-the-large (CITL), and the expected-to-observed (E:O) ratio. Internal validation was achieved through two complementary approaches: assessing performance in the randomly held-out validation cohort and applying bootstrap resampling with 1,000 iterations within the development cohort to evaluate model stability and estimate optimism-corrected calibration. Finally, a decision curve analysis (DCA) was conducted to assess the clinical utility and net benefit of the model across a range of threshold probabilities. All statistical analyses were performed using SPSS version 26.0 (IBM Corp., Armonk, NY, USA) and R version 4.1 (packages: rms, rmda, pROC, dplyr, tidyr, ResourceSelection, and mice). A two-sided *p* < 0.05 was considered statistically significant.

## 3 Results

### 3.1 Study cohort

Between 1 January 2023 and 1 June 2023, a total of 397 infants undergoing elective CPB surgery for congenital heart disease met the inclusion criteria and were included in the final analysis. Patients were randomly divided into a development cohort (*n* = 277) and a validation cohort (*n* = 120) in a 7:3 ratio. Following random allocation, there were no statistically significant differences in baseline demographics or perioperative variables between the development and validation cohorts (all *p* > 0.05). Baseline demographic, preoperative, intraoperative, and CPB-related characteristics of the two cohorts stratified by the occurrence of postoperative CDEs are summarized in Table 1. Early postoperative CDEs upon admission to the cardiac intensive care unit were observed in both cohorts: 50 infants (18.1%) in the development cohort and 17 infants (14.2%) in the validation cohort experienced CDEs.

**Table 1:** Preoperative and intraoperative patient characteristics.

Variables^a^	Development cohort (*n* = 277)		*p-*Value	Validation cohort (*n* = 120)		*p*-Value
	Non-CDEs (*n* = 227)	CDEs (*n* = 50)		Non-CDEs (*n* = 103)	CDEs (*n* = 17)	
Age (months)	4.4 (3.1, 7.2)	5.4 (3.2, 7.6)	0.566	4.4 (3.5, 7.0)	3.8 (2.1, 6.3)	0.168
Sex			0.949			0.246
Male	126 (55.5)	28 (56.0)		51 (49.5)	11 (64.7)	
Female	101 (44.5)	22 (44.0)		52 (50.5)	6 (35.3)	
BSA (m^2^)	0.33 (0.30, 0.38)	0.31 (0.28, 0.36)	0.028^b^	0.33 (0.28, 0.38)	0.28 (0.24, 0.31)	0.006^b^
MAP (mmHg)	46.3 (38.8, 52.6)	45.2 (36.3, 51.2)	0.299	46.7 (39.3, 52.7)	42.7 (36.3, 55.7)	0.322
HR (bpm)	138 (120, 150)	138 (127, 150)	0.442	140 (128, 155)	134 (121, 146)	0.306
SpO_2_ (%)	99 (98, 100)	99 (97, 100)	0.818	99 (97, 99)	99 (98, 100)	0.819
Anesthesia time (min)	156.5 (140.0, 179.0)	182.0 (153.0, 210.0)	<0.001^b^	155.0 (138.0, 185.0)	190.0 (165.0, 205.0)	0.002^b^
ASA grade			0.520			0.201
Ⅰ	0 (0.0)	0 (0.0)		0 (0.0)	0 (0.0)	
Ⅱ	2 (0.9)	0 (0.0)		1 (1.0)	0 (0.0)	
Ⅲ	187 (82.4)	45 (90.0)		90 (87.4)	12 (70.6)	
Ⅳ	38 (16.7)	5 (10.0)		12 (11.7)	5 (29.4)	
CPB temperature			<0.001^b^			0.216
>28 °C	208 (91.6)	35 (70.0)		93 (90.3)	13 (76.5)	
≤28 °C	19 (8.4)	15 (30.0)		10 (9.7)	4 (23.5)	
Preoperative HCT (%)	32.0 (29.0, 33.0)	32.0 (29.0, 35.0)	0.093	31.5 (29.5, 32.3)	33.0 (32.0, 35.0)	0.022^b^
Intraoperative HCT (%)	32.0 (28.0, 33.0)	25.5 (24.0, 30.0)	<0.001^b^	31.0 (28.0, 33.0)	30.0 (25.6, 32.0)	0.308
CPB time (min)	65.0 (52.0, 88.5)	90.0 (67.3, 115.0)	<0.001^b^	66.0 (49.5, 80.0)	88.0 (71.0, 120.0)	0.002^b^
Aorta clamping time (min)	35.0 (26.0, 48.0)	47.0 (36.0, 69.0)	<0.001^b^	35.0 (26.0, 45.0)	49.0 (33.0, 55.0)	0.020^b^
Number of cardiac surgeries			0.128			>0.999
Once	224 (98.7)	47 (94.0)		101 (98.0)	17 (100.0)	
More than once	3 (1.3)	3 (6.0)		2 (2.0)	0 (0.0)	
Surgical position			0.011^b^			0.073
Supine	166 (73.1)	45 (90.0)		72 (70.0)	16 (94.1)	
Prone	61 (26.9)	5 (10.0)		31 (30.0)	1 (5.9)	
RACHS-1 score			<0.001^b^			0.002^b^
<3	131 (57.7)	12 (24.0)		65 (63.1)	4 (23.5)	
≥3	96 (42.3)	38 (76.0)		38 (36.9)	13 (76.5)	
Postoperative hypothermia			0.212			>0.999
Yes	60 (26.4)	9 (18.0)		27 (26.2)	4 (23.5)	
No	167 (73.6)	41 (82.0)		76 (73.8)	13 (76.5)	
Prematurity			0.682			0.861
Yes	12 (5.3)	4 (8.0)		11 (10.7)	1 (5.9)	
No	215 (94.7)	46 (92.0)		92 (89.3)	16 (94.1)	
Cerebral perfusion			0.655			0.052
Yes	4 (1.8)	2 (4.0)		1 (1.0)	2 (11.8)	
No	223 (98.2)	48 (96.0)		102 (99.0)	15 (88.2)	

a Continuous data are shown as median (interquartile range, Q1, Q3) and categorical data as number (%).

b Statistically significant (*p* < 0.05).

### 3.2 Model development

Several perioperative variables were associated with postoperative CDEs in univariable analyses; however, not all remained independently associated after multivariable adjustment and backward selection, suggesting overlap or shared information among candidate predictors. Prespecified candidate variables were evaluated in univariable analyses and subsequently entered into the multivariable logistic regression model, including longer anesthesia time, lower CPB temperature management strategy, smaller body surface area, lower intraoperative hematocrit, longer CPB duration, surgical position, and RACHS-1 score ≥3 (Table 1).

**Figure 2:**
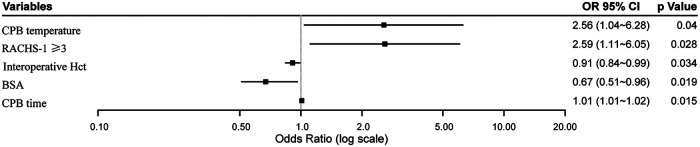
Forest plot of independent predictors of early postoperative cerebral desaturation events (CDEs). CPB temperature: ≤28°C vs. >28°C; CPB time: minutes; BSA: m²; Hct: %; RACHS-1: ≥3 vs. <3. CPB, cardiopulmonary bypass; Hct, hematocrit; BSA, body surface area; RACHS-1, Risk Adjustment for Congenital Heart Surgery.

A multivariable logistic regression analysis demonstrated that moderate/deep hypothermia during CPB, lower intraoperative hematocrit, prolonged CPB duration, higher surgical complexity (RACHS-1 score ≥3), and smaller body surface area were independently associated with an increased risk of postoperative CDEs (Figure 2).

**Figure 3:**
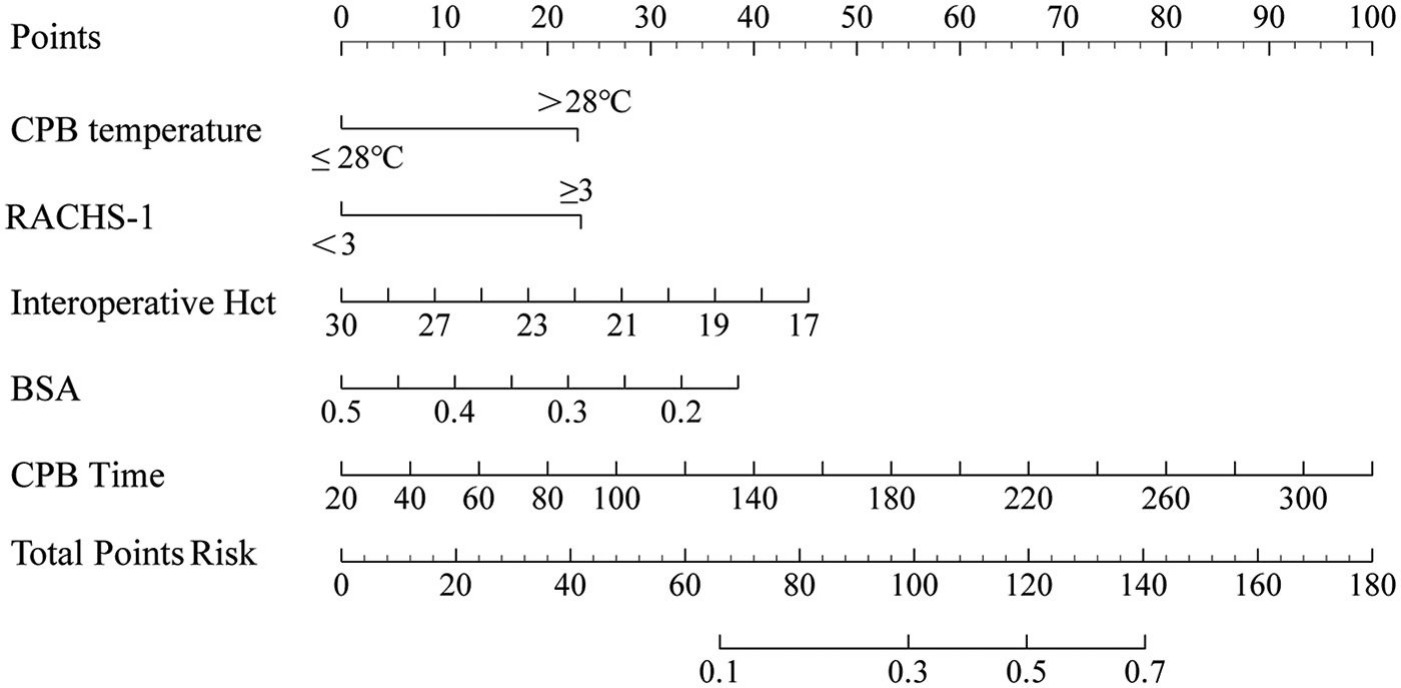
A nomogram to predict CDEs. The nomogram provides a visual point system based on the combination of perioperative predictors (CPB temperature, RACHS-1, intraoperative Hct, BSA, and CPB time) to estimate the probability of CDEs. To calculate the probability, add the points for each variable according to the scales to obtain the total points, then draw a vertical line from the total points scale to the bottom axis to read the corresponding probability of CDEs.

Based on these independent predictors, a nomogram was developed to estimate the individual probability of postoperative CDEs (Figure 3).

### 3.3 Model performance and internal validation

**Figure 4:**
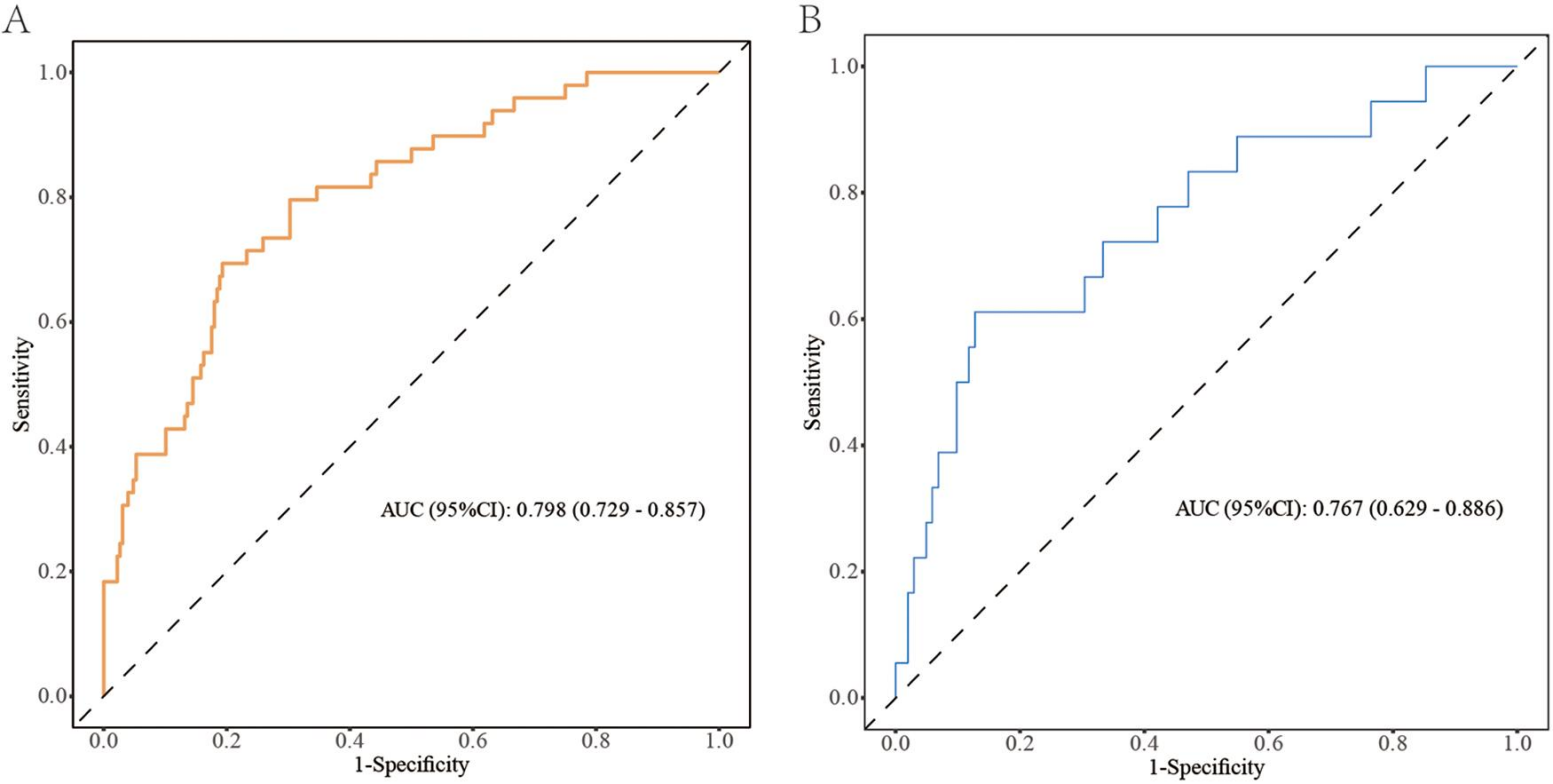
AUROC curve analysis of the development cohort **(A)** and validation cohort **(B)**. AUC, area under the curve.

**Figure 5:**
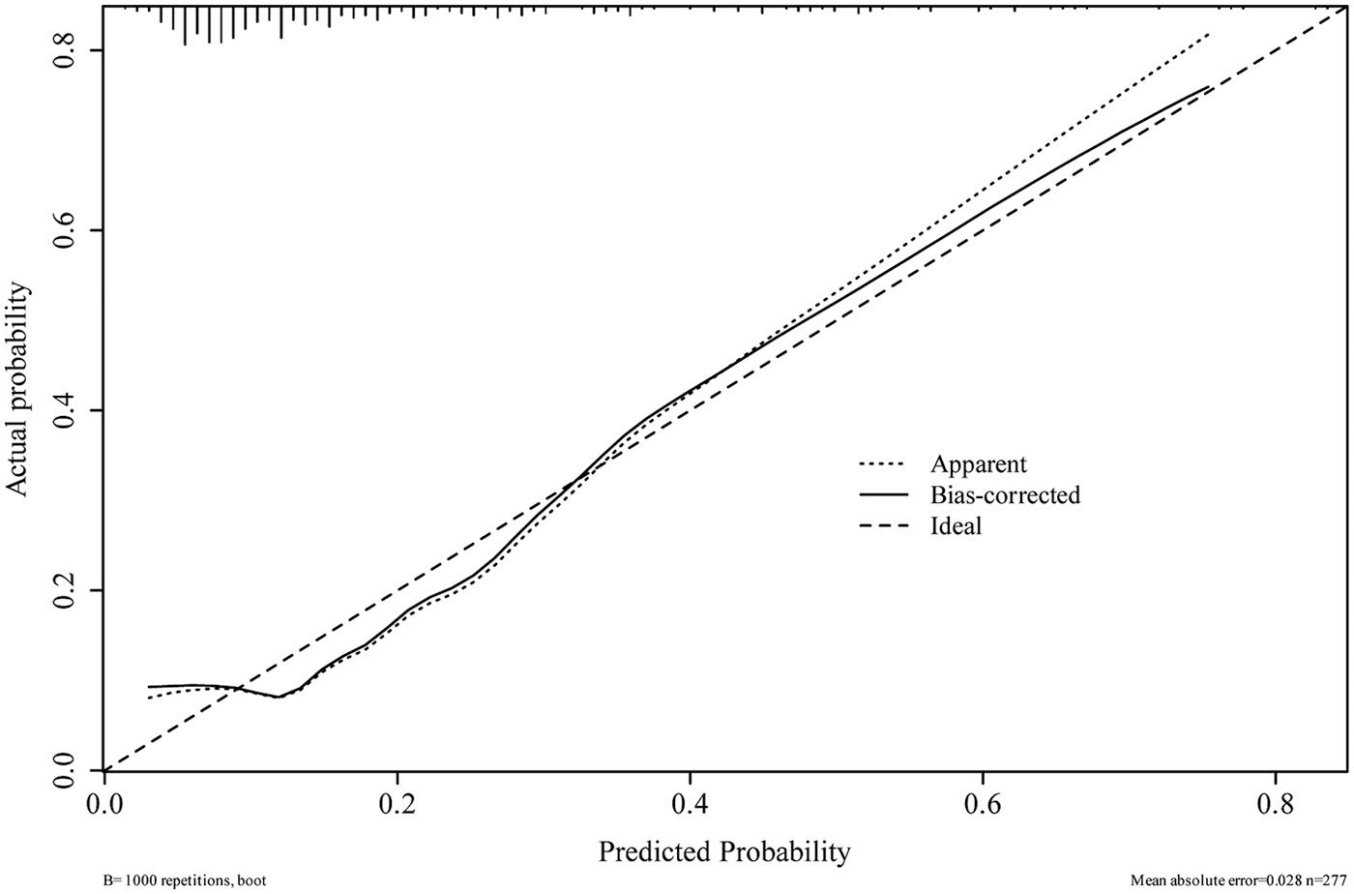
Internal calibration curves for the prediction model of postoperative CDEs. A perfectly calibrated model would generate a curve where the observed and predicted probabilities are completely matched along the 45° line (dashed line). The apparent calibration curve (dotted line) represents the performance of the model in the development cohort, whereas the bias-corrected curve (solid line) indicates the result after correction for optimism using 1,000 bootstrap resamples.

The *p*-value of the Hosmer–Lemeshow (H–L) goodness-of-fit test was 0.675. The C-statistic of the nomogram in the development cohort was 0.798 [95% confidence interval (CI), 0.729–0.857], demonstrating good discrimination (Figure 4A). The rates of sensitivity and specificity based on the AUROC curve were 67.4% and 84.2%, respectively. The apparent calibration curve showed good agreement between predicted and observed probabilities of postoperative CDEs, with a Brier score of 0.136, a CITL of 0.000, and an expected-to-observed (E:O) ratio of 1.00. In the validation cohort, the prediction model maintained good discriminatory performance. The C-statistic of the nomogram in the validation cohort was 0.767 (95% CI, 0.629–0.886) (Figure 4B). The sensitivity and specificity rates based on the AUROC curve were 61.7% and 78.9%, respectively. Calibration plot with an H–L type *χ*^2^ statistic *p*-value of 0.113 also showed good calibration in the validation cohort, with a Brier score of 0.141, a CITL of −0.129, and an E:O ratio of 1.04. After internal validation using 1,000 bootstrap resamples, the bias-corrected calibration curve also demonstrated satisfactory calibration across the clinically relevant range of predicted risk (Figure 5).

### 3.4 Decision curve analysis

**Figure 6:**
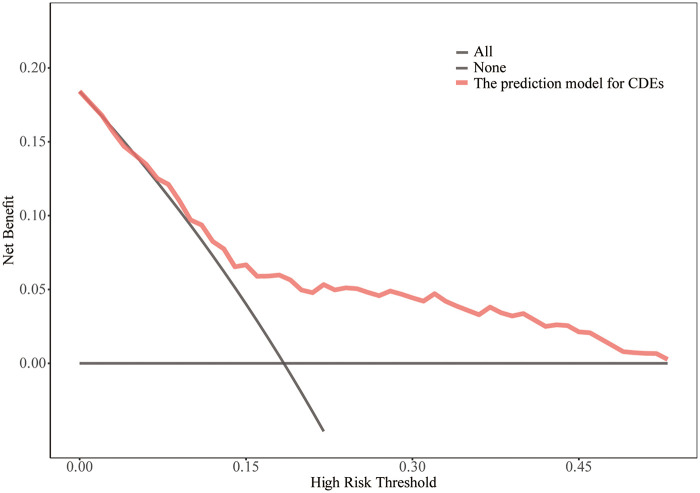
The DCA shows the clinical usefulness of the nomogram predicting postoperative CDEs. The Y-axis represents net benefit. The bold solid red line denotes the prediction model for CDEs, the solid grey line assumes that all patients experienced CDEs, and the horizontal black line assumes that none did. The DCA demonstrates that the nomogram provides a greater net clinical benefit across threshold probabilities from 0 % to 50 %.

The decision curve analysis demonstrated that, across a threshold probability range of approximately 0%–50%, the nomogram yielded a higher net benefit than either the treat-all or the treat-none strategy (Figure 6). These findings suggest that the model may be clinically useful for identifying infants at increased risk of early postoperative CDEs upon CICU admission.

## 4 Discussion

In this study, postoperative CDEs were observed in 16.88% of patients, with comparable incidence rates between the development cohort (18.1%) and the validation cohort (14.2%). These findings indicate that clinically significant CDEs remain common in infants undergoing cardiac surgery with cardiopulmonary bypass, highlighting the importance of early postoperative cerebral oxygen monitoring. Furthermore, five independent predictors of CDEs were identified. Based on these predictors, a novel nomogram was developed to estimate the individual risk of postoperative CDEs. The nomogram demonstrated good discrimination and calibration in both the development and validation cohorts. A DCA confirmed the clinical utility of the nomogram, indicating that clinical interventions guided by this predictive model would provide net benefit across a threshold probability range of 0%–50%.

Previous studies have identified several predictors associated with CDEs, including age, cardiopulmonary bypass duration, cerebral perfusion strategies, intraoperative hematocrit, and temperature management (13, 16, 17). Building on this literature, the present study demonstrates that the risk of early postoperative CDEs in infants is shaped by two complementary domains: baseline vulnerability (body surface area and RACHS-1 category) and modifiable intraoperative exposure (CPB temperature strategy, intraoperative hematocrit, and CPB duration). Prior literature has extensively examined prolonged CPB, hypothermic strategies, and hemodilution as determinants of impaired cerebral oxygenation and neurological risk in pediatric cardiac surgery (18, 22). Our results align with this body of evidence, while also indicating that these factors do not fully explain early postoperative CDEs in infants. The persistence of body surface area and RACHS-1 category as independent predictors after adjustment suggests that developmental reserve and complexity-related physiological stress add clinically relevant information beyond conventional perfusion metrics.

Within an infant-only cohort, body surface area appears to function as a more discriminative marker of developmental reserve than chronological age. Many pediatric studies identify younger age as a risk factor for perioperative neurological vulnerability (17, 23, 24). However, among infants under 1 year, age ranges are relatively compressed, while interindividual differences in somatic development, circulating blood volume, vascular caliber, and compensatory capacity remain substantial (22). Body surface area integrates several of these attributes and therefore plausibly captures susceptibility to abrupt changes in oxygen delivery, perfusion pressure, and thermal balance during the off-CPB transition (4). From a clinical standpoint, this interpretation supports using body surface area to trigger heightened vigilance for cerebral oxygenation instability, particularly when other risk factors such as prolonged bypass or deeper cooling are present (5).

The independent association between higher RACHS-1 category and postoperative CDEs extends the clinical relevance of surgical risk stratification beyond mortality and major morbidity. RACHS-1 has been validated as a predictor of postoperative outcomes in congenital heart surgery (25–27), but its linkage to early postoperative cerebral oxygenation has been less consistently explored. Higher RACHS-1 scores represent more complex repairs that entail greater operative manipulation (28, 29), higher likelihood of transient hemodynamic perturbations, and increased exposure to non-physiologic perfusion. These conditions plausibly increase cerebral oxygen supply–demand mismatch through periods of altered cerebral perfusion, reperfusion dynamics after rewarming (30), and a larger burden of inflammatory and microcirculatory disturbance (31).

To our knowledge, for model construction, this is one of the few studies to develop an infant-specific prediction model for early postoperative CDEs after congenital heart surgery with CPB. Notably, all variables included in the model were objective and routinely recorded perioperatively, enabling implementation without additional testing burden. A nomogram in the clinical context provides a visual point-based system to estimate an individual probability of postoperative CDEs, and in this study, it demonstrated good discrimination in both the development cohort (C-statistic 0.798, 95% CI 0.729–0.857) and the validation cohort (0.767, 95% CI 0.629–0.886). Calibration was satisfactory, with no evidence of miscalibration on the Hosmer–Lemeshow test in either cohort (*p* = 0.675 and *p* = 0.113, respectively), supporting agreement between predicted and observed risks. Furthermore, the decision curve analysis indicated that within a clinically relevant threshold probability range (approximately 0%–50%), management decisions informed by the model would yield a net benefit compared with treating all or treating none, underscoring its potential value as a decision-support tool for perioperative planning and early postoperative monitoring. As an early warning tool, this model may support strategies to reduce postoperative CDEs. Prospective studies are needed to determine whether early identification of high-risk infants before or at CICU admission can enable closer cerebral oxygenation monitoring and timely optimization of postoperative hemodynamic and respiratory management. Multicenter studies should further assess its impact on short-term outcomes and long-term neurodevelopment.

Several limitations in this study should be acknowledged. This was a single-center retrospective study, which may limit generalizability and is inherently subject to residual confounding. In addition, some potentially relevant perioperative variables were inconsistently documented in the medical records, such as the lowest intraoperative hematocrit and hematocrit before CICU admission, which may have introduced information bias. Although internal validation demonstrated stable model performance, external validation in independent cohorts is required before broader clinical application. Postoperative CDEs represent a physiological marker rather than a direct measure of long-term neurodevelopmental outcomes; therefore, studies incorporating longitudinal follow-up are needed to clarify clinical implications. Finally, this model is intended for risk stratification rather than diagnostic decision-making and should support, not replace, clinical judgment.

## 5 Conclusion

By analyzing infants undergoing congenital heart surgery with cardiopulmonary bypass, this cohort study identified five independent predictors of early postoperative CDEs, among which smaller body surface area and higher RACHS-1 category have been infrequently emphasized in previous research. Based on these predictors, we developed and validated a novel nomogram with reliable discrimination and calibration. By integrating baseline patient vulnerability and surgical complexity with perioperative factors, this model enables practical preoperative risk stratification and provides a quantitative framework to identify infants at high risk for early postoperative CDEs.

## Data Availability

The raw data supporting the conclusions of this article will be made available by the authors, without undue reservation.
