# Correction: A novel nomogram for predicting early postoperative cerebral desaturation events after congenital heart surgery

**DOI:** 10.3389/fsurg.2026.1865527

**Published:** 2026-05-18

**Authors:** Siyuan Wang, Weihong Xu, Bin Ji, Menglian Sun, Jianhu Huang, Jiwen Sun, Nanping Shen

**Affiliations:** 1 Department of Anesthesiology, Shanghai Children’s Medical Center, Shanghai Jiao Tong University School of Medicine, Shanghai, China; 2 Department of Thoracic and Cardiovascular Surgery, Shanghai Children’s Medical Center, Shanghai Jiao Tong University School of Medicine, Shanghai, China; 3 Department of Nursing, Shanghai Children’s Medical Center, Shanghai Jiao Tong University School of Medicine, Shanghai, China

**Keywords:** cardiac surgery, cardiopulmonary bypass, cerebral desaturation, near-infrared spectroscopy, perioperative neuroprotection, risk prediction nomogram

Authors Siyuan Wang and Weihong Xu were erroneously omitted as equal contributing authors in the author list. The following statement has been added: “†These authors contributed equally to this work and share first authorship”.

The original version of this article has been updated.

